# Antibacterial ZnO nanoparticle embedded polycaprolactone—polyhydroxybutyrate membranes for wound healing

**DOI:** 10.1007/s10856-026-07071-w

**Published:** 2026-05-26

**Authors:** Samanta Sam, Blessy Joseph, Neethu Ninan, Markos Negash Alemie, Krasimir Vasilev, Richard Bright, Sabu Thomas

**Affiliations:** 1 School of Energy Materials, Mahatma Gandhi University, Kottayam, Kerala India; 2 Business Innovation and Incubation Centre, Mahatma Gandhi University, Kottayam, Kerala India; 3 College of Medicine and Public Health, Flinders University, Bedford Park, SA Australia; 4 International and Inter-University Centre for Nanoscience and Nanotechnology, Mahatma Gandhi University, Kerala Kottayam, India

## Abstract

Wound management remains a significant clinical challenge, driving the development of multifunctional biomaterials that combine mechanical integrity, antibacterial activity, and biocompatibility. In this study, polycaprolactone–polyhydroxybutyrate (PCL–PHB) composite membranes incorporating zinc oxide nanoparticles (ZnO NPs) were fabricated via a solvent-casting approach and systematically optimised for wound-healing applications. ZnO NPs were incorporated at varying loadings (0.5–2 wt%), and the resulting membranes were characterised for morphology, chemical structure, crystallinity, surface wettability, mechanical performance, antibacterial efficacy, and cytocompatibility. FTIR, XRD, FESEM, and EDX analyses confirmed successful integration of ZnO nanoparticles within the PCL–PHB matrix, while FESEM observations suggested relatively uniform nanoparticle dispersion at lower ZnO concentrations. Incorporation of ZnO NPs significantly enhanced tensile strength and antibacterial performance, with optimal properties observed at intermediate nanoparticle loadings (1–1.5 wt%). Nanoparticle incorporation enhanced tensile strength from 2.0 MPa for the control membrane to a maximum of 4.8 MPa at 0.5 wt% ZnO loading. Higher ZnO concentrations led to nanoparticle aggregation and a decline in mechanical properties. The composite membranes exhibited pronounced antibacterial activity of about 90% against *Staphylococcus aureus* and moderate activity against *Pseudomonas aeruginosa* (32.5%). Importantly, all ZnO-containing membranes demonstrated high cytocompatibility with THP-1 monocyte-derived immune cells, maintaining cell viability above 80% and preserving normal cytoskeletal organisation. These findings highlight the importance of nanoparticle loading optimisation and nanoscale dispersion in the design of multifunctional antibacterial polymer nanocomposites for wound management.

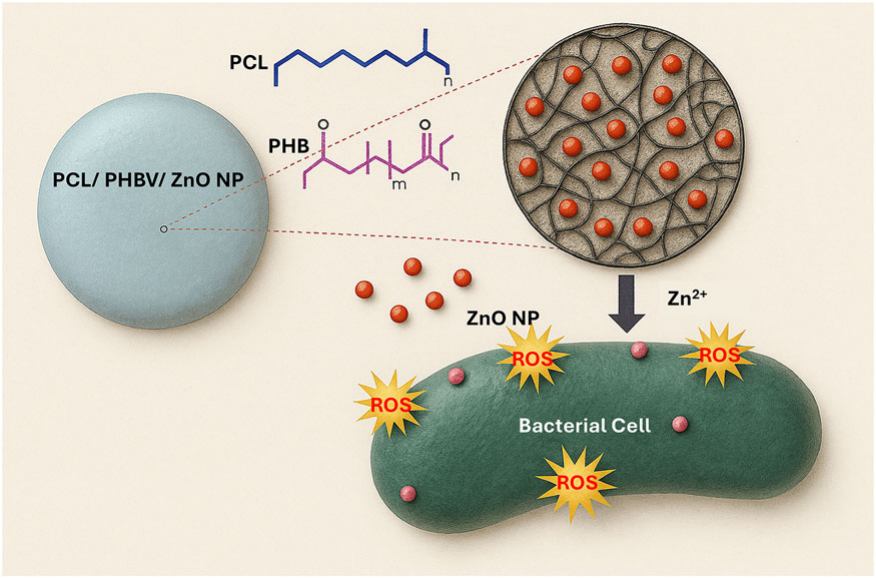

## Highlights

- Optimised ZnO loading (1–1.5 wt%) balances mechanical, antibacterial, and biological performance
- Tensile strength improved from ~2.0 MPa to ~4.8 MPa with ZnO incorporation
- Nanoparticle dispersion governs functionality
- Strong antibacterial activity ( ~ 90%) against *S. aureus* and moderate ( ~ 30%) against *P. aeruginosa*
- High cytocompatibility with THP-1 cells ( > 80% viability) and normal morphology

## Introduction

Biomaterials are increasingly being explored for diverse biomedical applications, particularly in wound healing, due to their biocompatibility and biodegradability [1, 2]. Biomaterials play a crucial role at multiple stages of wound healing, including cell proliferation, migration, and differentiation, offering new opportunities for tissue regeneration and repair. These materials often possess multifunctional properties, enabling them to support multiple phases of the wound-healing process [3]. Wound healing is a complex process that occurs naturally through a series of interconnected stages involving various initiators and mediators, including cytokines, macrophages, and fibroblasts [4, 5]. Combining biopolymers with wound-healing properties offers the potential to create matrices that stimulate and activate target cell responses, which are essential for the healing process. Among resorbable polymers, polycaprolactone (PCL) stands out due to its favourable solubility, low melting temperature (59–64 °C), and exceptional rheological and viscoelastic properties [6]. These characteristics have sparked significant research interest in its use in biomedical applications, including surgical sutures, tissue-engineering polymer membranes, and drug-delivery matrices [7]. The PCL chain consists of repeating hexanoate units linked by ester bonds. It is a biodegradable and biocompatible polymer approved by the U.S. Food and Drug Administration (FDA) for biomedical applications [8]. Its excellent tensile properties, versatility, biocompatibility, adaptability, availability, and cost-effectiveness make PCL a preferred polymeric material for use in various biomedical applications [9]. PCL has the unique ability to form blends with various other polymers, including polycarbonate and cellulose derivatives. Another biopolymer is poly(3-hydroxybutyrate) (PHB), a fully biodegradable and biocompatible polyester produced by bacterial fermentation of renewable resources such as cane sugar [10]. It has recently gained significant attention as a sustainable alternative to petroleum-based polymers. It is an isotactic linear thermoplastic composed of 3-hydroxybutyric acid units with the chemical structure [–O–CH–(CH₃)–CH₂–CO–]ₙ and was first discovered by Lemoigne in 1926 [11]. It is now manufactured on an industrial scale. It is the earliest and most widely used biopolymer in the family of polyhydroxyalkanoates (PHAs) [12]. Nevertheless, PHB has several inherent limitations for use as a polymer membrane, including brittleness, thermal instability in the molten state, a slow degradation rate, and the release of acidic degradation products [13]. Thus, the application of PHB-based materials can be expanded through chemical modification or copolymer formation to enhance hydrophilicity for biomedical use; however, such approaches often retain issues related to degradation and the intrinsic variability of biologically produced PHB [14]. Incorporating PCL was a rational strategy given its excellent mechanical performance, and the resulting PCL–PHB blends exhibited enhanced ductility and toughness, synergistically combining the high Young’s modulus of PHB with the intrinsic flexibility of PCL. This strategy underscores the common practice of blending PHB with other polymers to mitigate its inherent limitations. Singh et al. reported that blending PCL with PHB enhances flexibility and electrospinnability and provides controlled drug release [15]. Moreover, the PCL–PHB nanofibrous scaffolds exhibit tissue-mimetic elasticity, suitable degradability, and antimicrobial properties, enabling multidrug delivery for infected wound healing and improved biocompatibility [2]. Owing to the lack of inherent antimicrobial activity in both PCL and PHB, various nanostructured materials, including magnesium [16], copper [16], silver [17], titanium dioxide [18] and gold nanoparticles [19], have been incorporated into these matrices to enhance their antimicrobial properties. These nanoparticles (NPs) are particles in the 1–100 nm size range. They are unique because of their small size, large surface area-to-volume ratio, high carrier capacity, enhanced reactivity, and ease of surface property modification [17]. These NPs, when incorporated into a polymer matrix, are widely used in the biomedical field.

Recently, zinc oxide (ZnO) nanostructures have attracted significant research interest due to their easy accessibility, biocompatibility, low cost, and potential for surface modification with various functional groups. It is generally recognised as a safe (GRAS) material by the Food and Drug Administration and widely used in industrial and biomedical applications [18]. They exhibit exclusive physical and chemical properties, such as strong ultraviolet absorption and antimicrobial activity (pH range of 7–8), even in the absence of light. As a result, it is widely used in applications such as optical devices [19] and antimicrobial activity [20]. ZnO exhibits a notably stronger antibacterial effect against *Staphylococcus aureus* than other metal oxides. ZnO nanoparticles (ZnO NPs), in particular, are highly effective in improving the mechanical stability, barrier properties, and/or antibacterial properties of polymers such as PCL [21, 22]. The antimicrobial efficacy of ZnO NPs, which underpins their biomedical applications, is attributed to the production of reactive oxygen species (ROS) via electron-hole pair formation, a process influenced by particle size [23]. Felice et al. reported that higher ZnO concentrations promote early mineralisation, evidenced by elevated alkaline phosphatase activity, increased cell proliferation, and positive Alizarin Red S staining of calcium deposits. Furthermore, it was observed that the PCL degradation rate varies with ZnO concentration [24].

ZnO NP–reinforced PCL and PHB systems have been widely explored for antibacterial wound-dressing applications; however, most studies have focused primarily on antibacterial performance and on responses of fibroblasts or keratinocytes. Systematic optimisation of ZnO NP loading in blended PCL–PHB matrices, particularly in relation to nanoscale dispersion, polymer–nanoparticle interfacial interactions, and their combined effects on mechanical properties and surface wettability, remains insufficiently investigated. In addition, despite the critical role of monocyte-derived immune cells in early inflammatory regulation and wound healing, their interactions with ZnO-based polymer nanocomposites have received limited attention. In this context, the present study systematically evaluates the influence of ZnO NP concentration on the structural, mechanical, antibacterial, and immune-cell-compatibility of PCL–PHB nanocomposite membranes, providing design insights for the development of optimised multifunctional antibacterial wound-dressing materials.

## Materials and methods

### Reagents

All the chemicals and reagents were of analytical grade and used without further purification. PCL was purchased from Sigma-Aldrich with a molecular weight of 80,000 g/mol, a glass transition temperature of –60 °C, a melt flow index of around 7 g/10 min (160 °C/2.16 kg) and a specific gravity of 1.10 g cm^–3^. PHB was a kind gift from the Siberian Federal University. Chloroform was purchased from Merck EMPLURA^®^ ( > 99%). Spherical ZnO nanopowders were procured from Sigma-Aldrich ( < 100 nm particle size).

Streptomycin and penicillin were acquired from Life Technologies. Roswell Park Memorial Institute (RPMI) medium was purchased from Sigma-Aldrich (Australia). Foetal bovine serum (FBS) and Tryptone Soy Broth (TSB) were bought from Thermo Scientific and Oxoid. 4′,6-diamidino-2-phenylindole (DAPI), phalloidin, and BacLight^TM^ viability kit were bought from Thermo Fischer Scientific. Silicon wafers were acquired from ProSciTech, Australia. *Staphylococcus aureus* (*S. aureus)* (ATCC 25923) and *Pseudomonas aeruginosa (P. aeruginosa)* (ATCC 15692) were used for antibacterial testing. THP-1 monocyte cell lines were used for cell toxicity and immunofluorescence studies.

### Preparation of ZnO-incorporated PCL–PHB polymer membranes

PCL (8 wt.%) and PHB (3 wt.%) were separately dissolved in 8 mL and 10 mL of chloroform (CHCl₃), respectively, and then mixed after complete dissolution. Then 0.5 mL of castor oil was added, and the mixture was stirred for 2 h. The ZnO NPs were nanoscale in dimension, enabling high surface area–to–volume ratios and effective interfacial interactions within the polymer matrix. Different concentrations of ZnO (0.5, 1, 1.5, and 2%) were sonicated in 2 mL of CHCl_3_ for 15 min. This was then added to the parent sample and stirred overnight at 50 °C. The mixture was then sonicated for 15 min and cast onto a petri dish. The prepared samples were labelled: PHZ (0.5%), PHZ (1%), PHZ (1.5%), and PHZ (2%), each with a different concentration of ZnO NPs.

### Characterisation techniques

#### Field emission scanning electron microscopy (FESEM)

The surface morphology of the nanocomposites was analysed using FESEM (FEI Nova Nano SEM 450) at 5 kV and a working distance of 5 mm. The PCL, PHB, and ZnO-incorporated PCL–PHB polymer membranes were carefully sectioned, mounted on specimen stubs using double-sided adhesive tape, and coated with gold using a sputter coater. The average feature/domain size of each sample was measured from SEM images using ImageJ, version 1.54i (NIH, MD, USA).

#### Transmission electron microscopy (TEM)

A Transmission Electron Microscope from JEOL JEM 2100 working at an accelerating voltage of 100 kV was used to assess the average particle size and morphology of the ZnO NPs.

#### Attenuated total reflection Fourier transform infrared (ATR-FTIR)

ATR-FTIR spectra were recorded on a Perkin Elmer Spectrum Two FT-IR spectrometer equipped with a diamond crystal and scanned over 400–4000 cm^–1^ with a resolution of 4 cm^–1^. This was conducted to find the functional groups and study the interaction between ZnO and the PCL–PHB matrix.

#### X-ray diffraction (XRD) analysis

XRD analysis was performed to determine the crystallinity index of the sample. It was recorded on a Bruker AXS D8 Advance powder diffractometer with a scan speed of 2°/min and a scan range from 0 to 50° using Bragg-Brentano (θ, 2θ) geometry. The measurements were taken using Cu Kα radiation from an X-ray tube with a wavelength of 1.5406 Å.

#### Tensile properties

The tensile strength of the nanocomposite polymer membranes was evaluated using a Universal Testing Machine (Tinius Olsen H50KT, United States) in accordance with ASTM D 882–12. The initial gauge length was set to 40 mm, and a constant crosshead speed of 10 mm/min was applied. The experiments were conducted in triplicate, and the mean of the obtained values was reported.

#### Energy-dispersive X-ray spectroscopy (EDX) analysis

EDX spectra were coupled with FESEM, allowing in situ elemental analysis. Gemini 300, with an aperture size range of 10–300 µm, was used. EDX provides both qualitative and semi-quantitative data to verify elemental incorporation, detect impurities, and map elemental distribution.

#### Contact angle

The hydrophilicity and hydrophobicity of the samples were evaluated from sessile drop water contact angle measurements recorded using an RD-SDMO2 goniometer. The samples were positioned on a flat, solid platform, and 2 µL of MilliQ water was applied to each sample at three different positions. Images of droplets were captured using a CCD camera every 30 s. The ImageJ software with the DropSnake plugin was used to measure the contact angle from the acquired images. Samples were analysed in triplicate, and the mean ± SD of these values was tabulated.

### Antibacterial activity

The UV-sterilised samples were placed in 24-well plates, and 500 µL of TSB was added, followed by incubation for 4 h at 37 °C. They were then inoculated with *S. aureus* (ATCC 25923) and *P. aeruginosa* (ATCC 15692) at 1 × 10^5^ colony-forming units per mL (CFU/mL) in each well. They were incubated for 18 h at 37 °C. The samples were diluted, and their optical density was recorded in triplicate at 600 nm.

### Cell viability

THP-1 human monocytes were grown at 37 °C in Roswell Park Memorial Institute (RPMI) medium containing 10% FBS and 1% Penicillin/Streptomycin in a 5% CO_2_ incubator. All samples were UV-sterilised, placed in sterile 24-well plates, and immersed in RPMI medium alone to collect leachables or extracts, in accordance with ISO 10993-5. Subsequently, 1 × 10 ^5^ THP-1 cells were seeded into 24-well plates and allowed to form a monolayer overnight at 37 °C, 95% humidity and 5% CO_2_. Next, the THP-1 cells were treated with 100 ng/mL PMA to differentiate into macrophage-like cells (dTHP-1) and incubated overnight at 37 °C, 95% humidity and 5% CO_2_, resulting in an adherent phenotype.

The medium was then replaced with collected leachables and incubated overnight. The wells were then incubated with 10 µL of 5 mg/mL 3-(4,5-dimethylthiazol-2-yl)-2,5-diphenyltetrazolium bromide (MTT) at 37 °C, 95% humidity and 5% CO2, for 4 h. The MTT solution was removed, and 200 μL of DMSO was added to solubilise the formazan crystals for 4 h. The absorbance of the plate was measured using a BioTek Synergy HTX Multimode microplate reader (Agilent Technologies, CA, USA) at 570 nm.

### Cytoskeletal and nuclear staining of dTHP-1 cells

Differentiated THP-1 cells (1 × 10⁵ cells/mL) were seeded into 24-well plates and incubated overnight at 37 °C and 5% CO_2_ to allow monolayer formation. Collected leachables from polymer membranes, then replaced the medium, and cells were incubated at 37 °C and 5% CO_2_ for 24 h. After incubation, the media was discarded, and the cells were fixed for 30 min with 4% Formaldehyde. Triton X-100 in phosphate-buffered saline (PBS) was used to permeabilise the cells, followed by blocking with 5% bovine serum albumin. The fixed cells were rinsed three times with PBS. The cells were stained with DAPI for 5 min and with Phalloidin (diluted 1:1000) for another 5 min. The cell images were captured using a ZEISS LSM 880 microscope (Zeiss, Germany).

### Statistical analysis

All quantitative data are presented as mean ± standard deviation (SD) from three independent experiments unless otherwise stated. Statistical analyses were performed using GraphPad Prism software, version 10.2.0 (392) (GraphPad Software, San Diego, CA, USA). Comparisons among multiple groups were performed using one-way analysis of variance (ANOVA), followed by Tukey’s post hoc test to identify statistically significant differences between groups. A *p* value of less than 0.05 was considered statistically significant.

## Results

### Fabrication of ZnO NP- PCL–PHB nanocomposites

**Scheme 1:**
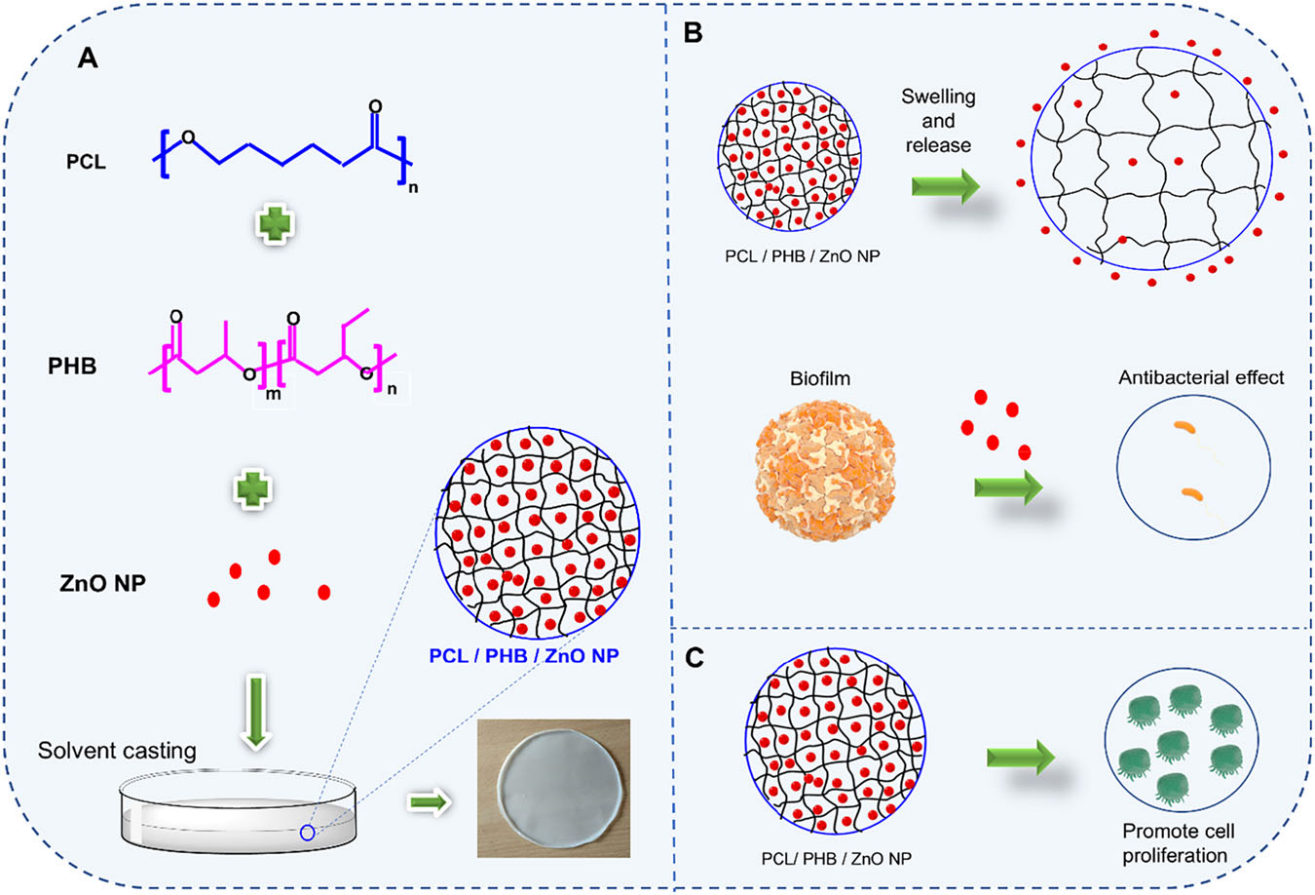
Schematic representation of (**A**) fabrication of PCL–PHB polymer membranes reinforced with ZnO NP, with (**B**) antibacterial effect and (**C**) biocompatibility

Functionalised PCL–PHB composite membranes containing ZnO NPs were successfully prepared using the solvent-casting method (Scheme 1). The membranes were formed by dissolving the polymers in chloroform, incorporating ZnO NPs at different loadings, and allowing solvent evaporation to produce structured films. The resulting samples included the control PCL–PHB membrane and ZnO-containing membranes with 0.5%, 1%, 1.5%, and 2% ZnO.

### Characterisation of the nanocomposite membranes

#### FTIR analysis

**Fig. 1:**
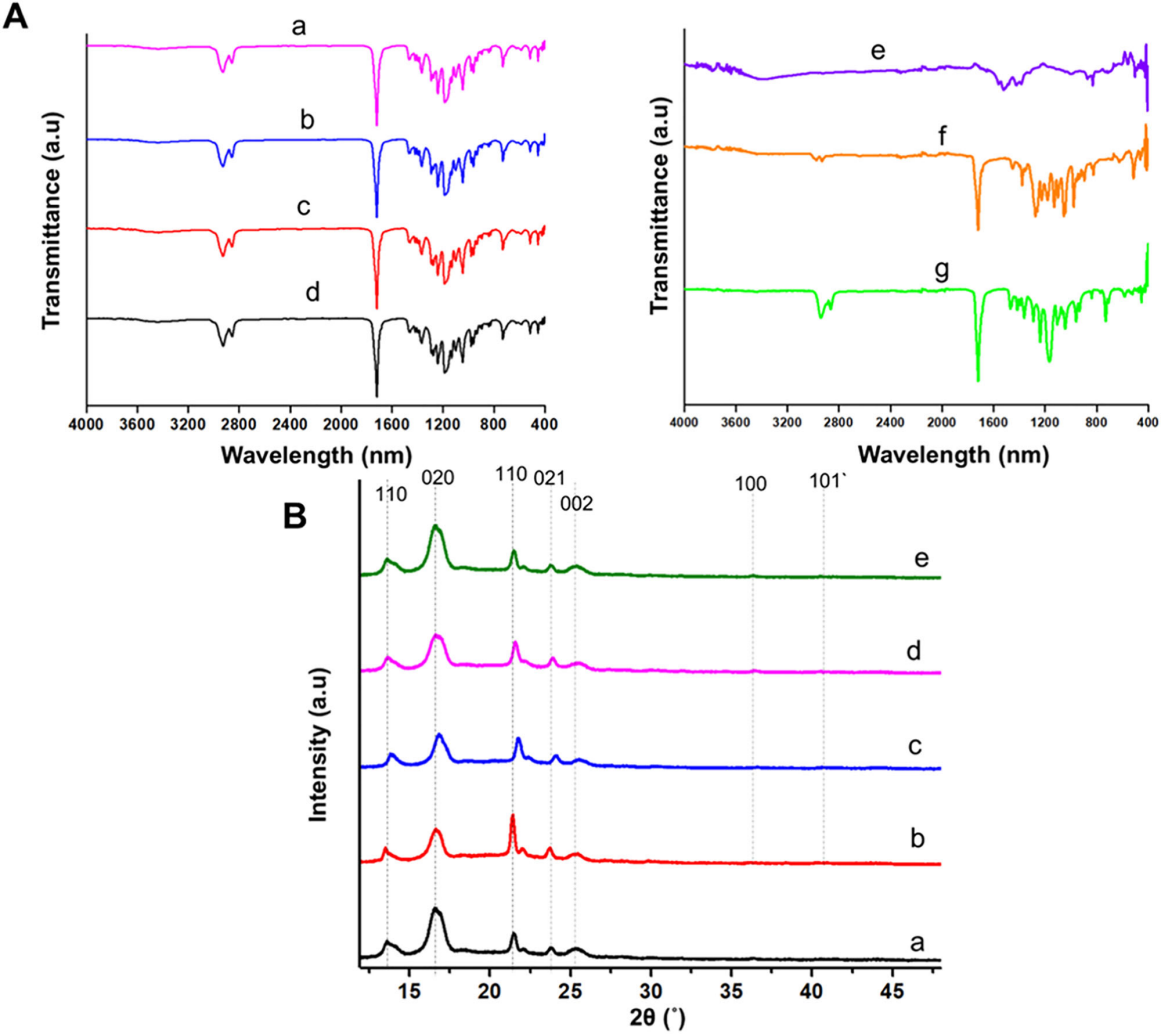
**A** FTIR spectra of (a) PHZ (0.5%), (b) PHZ (1%), (c) PHZ (1.5%), (d) PHZ (2%), (e) ZnO, (f) PHB, and (g) PCL. **B** XRD spectra of (a) Control, (b) PHZ (0.5%), (c) PHZ (1%), (d) PHZ (1.5%), and (e) PHZ (2%)

FTIR spectra were obtained for neat PCL, PHB, and ZnO NPs, as well as for PCL–PHB composite polymer membranes containing varying concentrations of ZnO NPs, as shown in Fig. 1A. The FTIR spectrum of ZnO NPs displays a broad absorption band in the region of ~430–500 cm⁻¹, which corresponds to the Zn–O stretching vibrations, characteristic of metal oxide frameworks [25]. The FTIR spectra of the PCL–PHB blends reveal the characteristic absorption bands associated with the functional groups of both polyesters. The bands observed in the range of 2940–2865 cm⁻¹ correspond to the asymmetric and symmetric stretching vibrations of methylene (–CH₂–) groups, which are present in the aliphatic backbones of both PCL and PHB. A prominent absorption peak near 1720.5 cm⁻¹ is assigned to the ester carbonyl (C = O) stretching vibration, common to both polymers, indicating the presence of ester linkages in the blend [26]. Although this band is prominent in both materials, its overlap in the blend prevents it from distinguishing between the individual polymers. The C–O–C asymmetric and symmetric stretching vibrations of PCL are evident at 1293 and 1240 cm⁻¹ [27]. While a nearby band at 1277.89 cm⁻¹ is more specific to PHB [28], providing a distinguishing spectral feature. Additional peaks in the 1170–1100 cm⁻¹ range, arising from C–C and C–O stretching vibrations, further support the presence of PCL [29]. Similarly, the absorption bands between 1050 and 1200 cm⁻¹ correspond to C–O and C–C stretching in PHB [28].

#### XRD analysis

The XRD spectra of the ZnO NPs dispersed in the PCL–PHB matrix are shown in Fig. 1B, which reveals distinct crystalline features confirming their structural characteristics. The diffraction peaks observed in the range of 13°–22° (2θ) were ascribed to the crystalline regions of the PCL and PHB matrix. Specifically, PHB exhibits characteristic reflections at approximately 13.5°, 17°, 20°, and 22° [29], characteristic of its orthorhombic crystalline structure, while PCL shows characteristic sharp peaks at 21.3°, corresponding to the (110) plane, and at 23.6°, corresponding to the (200) plane [30]. These sharp, well-defined peaks confirm the semi-crystalline nature of the polymer blend, indicating an orthorhombic polymer structure [31]. The PCL–PHB blend membrane exhibited characteristic diffraction peaks of both polymers, without new reflections, confirming physical blending rather than chemical interaction. Slight peak attenuation and broadening indicate partial inhibition of crystallisation and reduced crystallite size due to restricted chain mobility. In addition, the presence of minor peaks at 31.7° and 34.4° corresponds to the (100) and (002) planes of hexagonal wurtzite ZnO [32] and a small peak at 47° corresponding to the (102) plane, as referenced by JCPDS card no. 36–1451 confirms the successful incorporation of ZnO NPs in the matrices of PHZ (0.5%), PHZ (1%), PHZ (1.5%), and PHZ (2%), without structural degradation. The relatively low intensity of these peaks is attributed to the low ZnO content incorporated into the composite. Additionally, minor peak shifts and intensity variations were observed in the nanocomposite membranes, indicating that ZnO acted as a heterogeneous nucleating agent, altering polymer crystallisation with increasing nanoparticle loading. The characteristic peaks of PCL and PHB remain distinctly observable even with increasing concentrations of ZnO NPs, indicating their uniform dispersion within the polymer matrix. Overall, the XRD analysis indicated that the characteristic crystalline phases of PCL, PHB, and ZnO were preserved within the composite membranes, while interfacial interactions modulated the degree of crystallinity and structural organisation.

#### Surface morphology

**Fig. 2:**
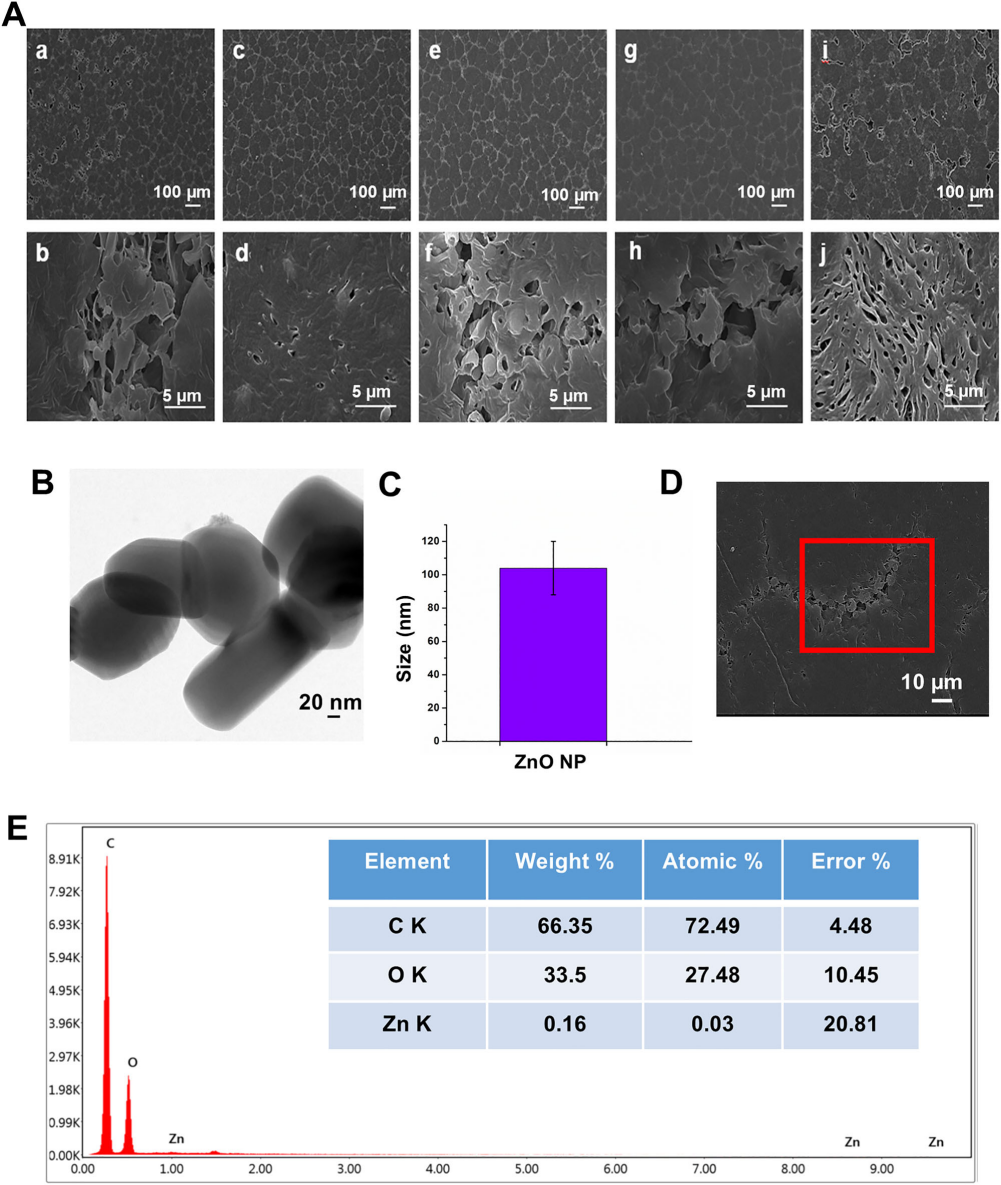
**A** Representative FESEM images of (a-b) Control, (c-d) PHZ(0.5%), (e-f) PHZ (1%), (g-h) PHZ (1.5%), and (i-j) PHZ (2%) at low and high magnifications; **B**, **C** TEM images of ZnO NPs and the particle size distribution. **D** FESEM image highlighted with the region that was scanned for EDX analysis (**E**), EDX spectra of a representative sample, PHZ (2%), and the elemental composition

From the TEM images in Fig. 2B, C, it is evident that the average particle size of ZnO NPs was 104 ± 16 nm. The surface morphology of solvent–cast PCL–PHB polymer membranes was evaluated using FESEM analysis. A non-uniform surface with irregularities was observed in the case of the PCL–PHB polymer membrane (control) due to phase separation after solvent evaporation. Once ZnO NPs were added from low to high concentrations, noticeable differences were found in the surface morphology. The nanocomposites exhibited a well-connected morphology with several domains uniformly distributed, likely due to improved dispersion of ZnO NPs and effective interactions with the polymer blends. However, a loss of surface uniformity was observed in the case of PHZ (2%) due to agglomeration of ZnO NPs (Fig. 2A). The observed morphological features further suggest good interfacial compatibility and adhesion between the PCL matrix and the ZnO nanofillers. FESEM images acquired from multiple random regions suggested relatively uniform nanoparticle dispersion at lower ZnO concentrations, while localised agglomeration became more evident at 2 wt% ZnO.

#### EDX analysis

The EDX spectra (Fig. 2E) confirmed the presence of ZnO NPs dispersed within the PCL–PHB matrix, as evidenced by a characteristic zinc emission peak near 1 keV, corresponding to its binding energy [33]. The relatively low intensity of the Zn signal is attributed to the low concentration of ZnO NPs incorporated into the composite. Additionally, the detected signals for carbon (C) and oxygen (O) are primarily attributed to the organic polymer components, PCL and PHB, constituting the matrix.

#### Surface wettability

**Fig. 3:**
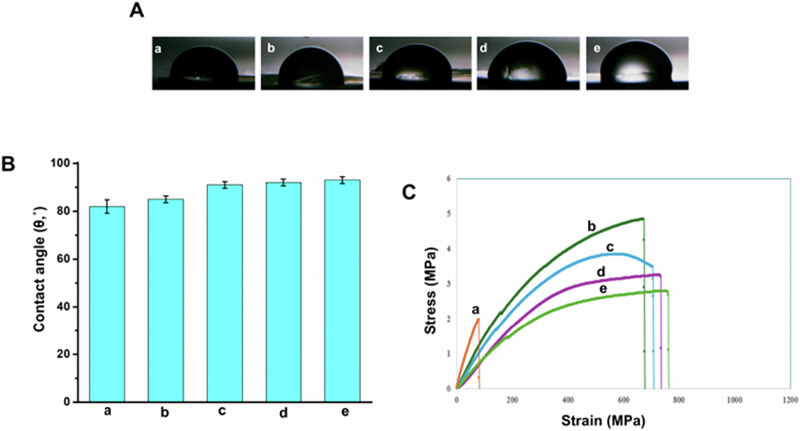
**A** Drop images and **B** Water contact analysis of (a) Control, (b) PHZ(0.5%), (c) PHZ (1%), (d) PHZ (1.5%), and (e) PHZ (2%). Data plotted as mean ± SD and *n* = 3. **C** Stress-strain graph of (a) Control, (b) PHZ (0.5%), (c) PHZ (1%), (d) PHZ (1.5%), and (e) PHZ (2%)

Water contact angle measurements are shown in Fig. 3A, B. The control PCL–PHB membrane exhibited a contact angle of 82 ± 2°. Following ZnO incorporation, the contact angle increased progressively from 85 ± 1.4° to 93 ± 1° with increasing nanoparticle concentration.

#### Mechanical properties

Stress–strain curves are shown in Fig. 3C. The control membrane exhibited the lowest tensile strength and elongation at break, with values of 2.0 MPa and 110%, respectively. Incorporation of ZnO improved both parameters. The 0.5% ZnO membrane showed the highest tensile strength at 4.8 MPa and an elongation at break of 650%. The 1% and 1.5% membranes showed tensile strengths of approximately 3.8 and 3.2 MPa, respectively, with elongation values of 690% -730%. The 2% membrane exhibited reduced tensile strength (2.8 MPa) but the highest elongation at break (770%).

### Antibacterial activity

**Fig. 4:**
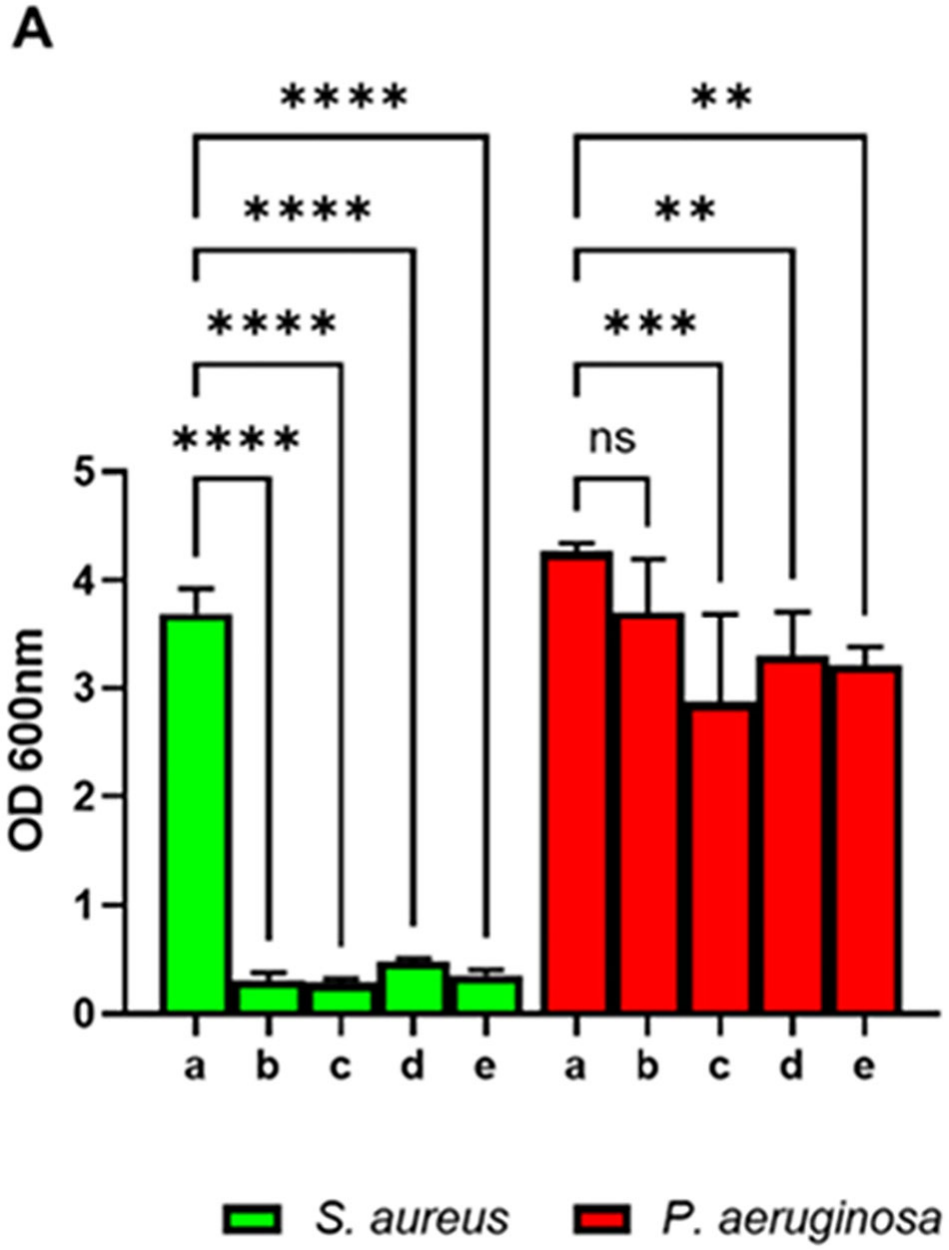
**A** Antibacterial properties of (a) PH, (b) PHZ (0.5%), (c) PHZ (1%), (d) PHZ (1.5%), and (e) PHZ (2%) composite polymer membranes. Data plotted as mean ± SD, *n* = 3, ** *p* < 0.01, *** *p* < 0.001, **** *p* < 0.0001, and ns is non-significant

The antibacterial performance of the membranes against *S. aureus* and *P. aeruginosa* is shown in Fig. 4A. ZnO-containing membranes reduced bacterial growth for both strains compared with the control. The reduction was more pronounced against *S. aureus* (approximately 90%, *p* < 0.0001 for all treatments) than against *P. aeruginosa* (approximately 32.5%, *p* < 0.01 for treatments c–e).

### dTHP-1 viability and cytoskeletal organisation

**Fig. 5:**
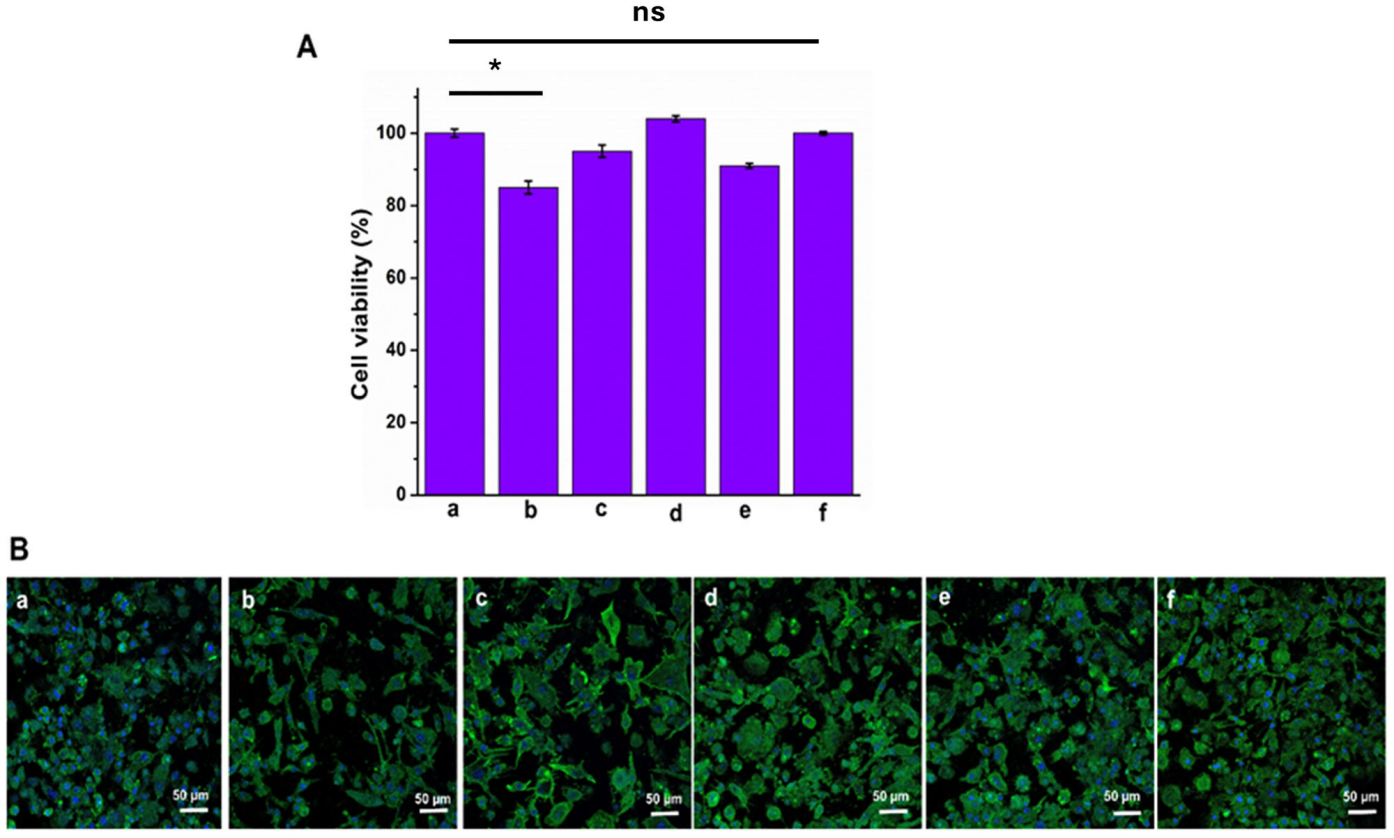
**A** MTT cell viability, plotted as mean ± SD, *n* = 3, * p < 0.05, and ns represents non-significance. Statistical comparisons were performed against the unexposed control group (a) using one-way ANOVA followed by Tukey’s post hoc test (*p* < 0.05). **B** Confocal images of THP-1 monocyte-derived cells. (a) Unexposed cells and those exposed to (b) PH, (c) PHZ (0.5%), (d) PHZ (1%), (e) PHZ (1.5%), and (f) PHZ (2%) composite polymer membranes. The green fluorescence represents the actin cytoskeleton, while the blue fluorescence corresponds to the cell nuclei stained with DAPI

Cell viability results are shown in Fig. 5A. (a) Unexposed cells and those exposed to (b) PH, (c) PHZ (0.5%), (d) PHZ (1%), (e) PHZ (1.5%), and (f) PHZ (2%). All groups maintained high cell viability ( > 85%), indicating good cytocompatibility of the fabricated membranes. No significant reduction in cell viability was observed compared with the control (*p* > 0.05), except for sample b. The ZnO-containing membranes showed slightly higher viability than the control membrane. Confocal images (Fig. 5B) showed that dTHP-1 monocyte-derived cells maintained normal morphology, with visible nuclei and organised actin cytoskeleton, across all membrane groups. Interestingly, the viability of the polymer membranes increased upon incorporation of ZnO NPs.

## Discussion

The present study demonstrates that incorporation of ZnO NPs into PCL–PHB membranes which substantially influenced the structural, surface, mechanical, antibacterial, and biological properties. FTIR and XRD analysis confirmed the successful integration of ZnO NPs into the polymer matrix, while preserving the characteristic features of both PCL and PHB. The lack of new major peaks associated with chemical bond formation suggests that ZnO was primarily physically dispersed within the blend rather than covalently interacting with the polymer chains.

Morphological observations support this interpretation. FESEM showed that low-to-intermediate ZnO loadings promoted a more connected, uniform surface morphology, whereas 2 wt% ZnO resulted in visible nanoparticle aggregation. This indicates that nanoscale dispersion was maintained at lower loadings but became less stable at higher concentrations. Such aggregation is important because it affects not only morphology but also functional performance [34–36].

The surface wettability data showed a modest increase in contact angle with increasing ZnO concentration. Although ZnO is often associated with hydrophilic behaviour [18, 23], in this system, the increased roughness and altered polymer surface organisation appear to have produced a slightly more hydrophobic surface. For wound-dressing applications [37], this may still be acceptable, as a balanced surface can assist in moisture control while maintaining cell compatibility [38].

Mechanical testing showed that ZnO had a strong reinforcing effect at low and moderate concentrations. The most pronounced improvement in tensile strength was observed at 0.5 wt% ZnO, indicating efficient stress transfer between the polymer matrix and well-dispersed nanoparticles [34]. At 1% and 1.5% ZnO, mechanical performance remained improved relative to the control, although tensile strength declined slightly from the 0.5 wt% maximum. At 2 wt%, tensile strength decreased further, which is consistent with the FESEM evidence of agglomeration [34]. These findings indicate that there is an optimal nanoparticle concentration range beyond which reinforcement benefits are lost because aggregates act as stress-concentrating defects [35].

The antibacterial data further support the functional role of ZnO in the composite membranes. All ZnO-containing samples inhibited bacterial growth, with a much stronger effect against *S. aureus* than against *P. aeruginosa*. This difference is consistent with the structural differences between Gram-positive and Gram-negative bacteria [39, 40]. The greater susceptibility of *S. aureus* is likely due to the absence of an outer lipopolysaccharide membrane, which, in Gram-negative bacteria, can reduce the penetration of Zn²⁺ ions and limit oxidative damage. The antibacterial activity is therefore likely mediated by a combination of Zn²⁺ release and reactive oxygen species generation at the particle–bacteria interface [23, 41].

Importantly, improved antibacterial activity was achieved without compromising immune-cell compatibility. All membranes maintained THP-1 viability above 80%, and fluorescence imaging showed preserved cytoskeletal organisation and normal cell morphology. These findings suggest that ZnO incorporation at the tested concentrations did not cause overt cytotoxicity in monocyte-derived immune cells [23]. This is particularly relevant because immune cells play a major role in early wound healing and biomaterial-associated inflammatory responses [42, 43].

Taken together, the results indicated that intermediate ZnO concentrations, particularly around 1–1.5 wt%, provide the best overall balance of nanoparticle dispersion, antibacterial performance, and cytocompatibility, while 0.5 wt% gives the strongest mechanical reinforcement. Therefore, the optimal loading depends on whether the priority is maximum tensile strength or balanced multifunctional wound-healing performance. Overall, this study highlights nanoparticle dose optimisation as a key design parameter in the development of multifunctional PCL–PHB-based wound-dressing membranes [34, 35].

Despite these promising findings, several limitations should be acknowledged. The antibacterial assessment was based on OD₆₀₀ screening and would be strengthened by confirmatory CFU counting and live/dead staining. Likewise, mechanistic studies such as Zn²⁺ release profiling and ROS quantification would help clarify how ZnO mediates antibacterial activity and influences cell behaviour. Long-term stability under physiological conditions and more detailed nanoparticle dispersion analysis were also not included. Future work should therefore evaluate these membranes in more complex wound models, including in vivo full-thickness or diabetic wound settings, and examine inflammatory signalling pathways to better establish translational potential.

## Conclusion

This study demonstrates that controlled incorporation of ZnO NPs into PCL–PHB membranes can effectively enhance mechanical performance, antibacterial activity, and immune-cell compatibility. Structural analyses confirmed successful ZnO incorporation within the polymer matrix, while morphological observations showed that nanoparticle dispersion strongly influenced the membrane performance. Mechanical testing revealed improved tensile properties at lower ZnO concentrations, whereas higher loadings promoted nanoparticle aggregation. The composite membranes also exhibited antibacterial activity, particularly against *S. aureus*, likely associated with Zn²⁺ release and ROS-mediated bacterial damage. Importantly, all membranes maintained high THP-1 cell viability and preserved cell morphology, indicating good cytocompatibility. Overall, intermediate ZnO concentrations provided the most balanced multifunctional performance, highlighting nanoparticle dose optimisation as an important design consideration for antibacterial wound-dressing materials. Future studies should investigate the underlying antibacterial mechanisms and evaluate the membranes in more clinically relevant wound-healing models.

## Data Availability

The data supporting the findings of this study are available from the corresponding author upon reasonable request. Public sharing of the data is restricted due to privacy and institutional constraints.
